# Television and eating: repetition enhances food intake

**DOI:** 10.3389/fpsyg.2015.01657

**Published:** 2015-11-03

**Authors:** Utsa Mathur, Richard J. Stevenson

**Affiliations:** Department of Psychology, Macquarie University, Sydney, NSW, Australia

**Keywords:** television, content, eating, environment, female

## Abstract

Some studies find that eating with TV increases food intake while others do not. Some of this variability may reflect the engagingness of what is being watched (i.e., content). To test this we varied engagingness by manipulating content familiarity. Female participants undertook two sessions. In the “Different” session they watched two different episodes of the comedy *Friends*, with snack food presented during the second episode. In the “Same” session they viewed another *episode* of *Friends* twice in succession, with snack food presented during the second repeat showing. The three episodes of *Friends* used here were fully counterbalanced, so overall the only difference between the “Same” and “Different” sessions was whether the content of the second show was familiar or novel. As expected, 14% less was eaten in the “Different” session, suggesting that novel and presumably more engaging content can reduce intake relative to watching familiar and presumably less engaging content. These findings are consistent with the idea that the engagingness of TV can differentially affect food intake, although boredom or irritability resulting from repeat viewing might also explain this effect.

## Introduction

Although eating with TV, relative to without, has been found to increase food intake (e.g., Blass et al., 2006; Hetherington et al., 2006; Braude and Stevenson, 2014), a number of studies have failed to find this effect (e.g., Martin et al., 2009; Peneau et al., 2009; Chapman et al., 2014). One reason for this variability may be that TV can affect eating in several ways. This could include altering mood (e.g., Yeomans and Coughlan, 2009; Groesz et al., 2012; Bongers et al., 2013), priming food intake via adverts or from watching others eat or cook (e.g., Harris et al., 2009; Bodenlos and Wormuth, 2013), or by drawing attention away from cues that may signal the end of a meal (e.g., Braude and Stevenson, 2014). In this last mentioned case, the idea that activities concurrent with eating (e.g., TV, computers, conversation, radio) distract attention away from food intake has been suggested in a number of studies (e.g., Brunstrom and Mitchell, 2006; Hetherington et al., 2006; Ogden et al., 2013). While no study has as yet quantified the level of distraction required to reduce or enhance food intake, the general premise has been that relative to a baseline of no concurrent activity, greater food intake may occur with *some* distraction, while too much may reduce eating to or even below baseline.

Apart from specifying precisely what constitutes different levels of distraction, there are at least two further problems with a distraction account of TVs effect on food intake. The first concerns evidence that drawing attention away from eating—distraction—affects food intake. Favorable evidence has been inferred from two types of procedure. In one, a task is presented concurrently with eating and this is then contrasted to the effects of presenting another different task also concurrently with eating. The dependent variable is the amount eaten, and the impact of distraction can be inferred by differences in intake between the two tasks [e.g., driving (more distracting) vs. TV, (less distracting); Ogden et al., 2013]. In the other, the task remains the same (e.g., viewing TV), but what varies is the content (i.e., the material presented during TV viewing). Thus, in this case, the effects of distraction can be inferred from alterations in content [e.g., boring (less distracting) vs. funny (more distracting); Chapman et al., 2014].

In the most recent TV content manipulation study (noting that this was not a test of the distraction account) Chapman et al. (2014) using a within-subject design, had participants watch a boring TV show, an engaging TV show or read a boring text, all while eating. Chapman et al. (2014) found that the boring TV show was associated with greater food intake than the comedy show, with the text condition (baseline) falling in between. Consistent with the distraction account, the comedy show may have been sufficiently engaging to slow or interrupt eating (relative to baseline), while the boring-TV condition may have been sufficiently distracting only to interfere with interoceptive cues to meal termination (e.g., Braude and Stevenson, 2014). The problem with this interpretation, and the interpretations of other studies that vary task or content (e.g., Mittal et al., 2010; Ogden et al., 2013) is that distraction is not manipulated independently from task or content. Consequently, we cannot be sure that any effects on food intake stem from variation in engagement (i.e., distraction) or from differences in content.

A second reason to question the distraction account comes from a recent study by Tal et al. (2014). Here, participants were randomly assigned to one of three snacking with concurrent TV groups. In one group participants watched a TV talk show, in another they viewed a fast paced action movie clip and in a third, they watched the same clip but without sound. Contrary to what one might expect from a distraction account the highest food intake was observed in the action movie clip with sound, with the lowest intake in the talk show. To the extent that the action movie clip was engaging—and it involved significantly greater number of changes in visual and auditory content than the other clips—this should according to the distraction account have led to a reduction in food intake relative to the other two conditions, as the movie presumably fully engaged participant’s attention. While again this experiment was not a formal test of the distraction account, it does suggest that the content of the TV show may independently affect food intake.

An important addition to the distraction, eating and TV literature would be to try and manipulate distraction independent of content. Following such a manipulation, any effect on food intake would be specific to the effects of distraction (or relatedly to differences in boredom, inattention or engagement), rather than to content *per se* (or at least within the genre from which the content was drawn). The experiment described here attempted this by varying content familiarity, with the idea being that novel content would be more engaging and distracting relative to familiar content (see Table 1 for design). There are two key features to this design. The first is its manipulation of familiarity. Participants snack on one occasion with a TV comedy show they have just seen before (Same session in Table 1) and on another occasion with a novel episode (Different session in Table 1) drawn from the same TV series (*Friends*). The second key feature of the design is that across the two within-participant sessions (Same vs. Different), content is equated. So, of the three TV episodes of *Friends* used here, each is as likely to serve as episode X in the Same session as it is to serve as episode Y or Z in the Different session (see Table 1). Thus any difference in food intake between the Same and Different session cannot be attributed to differences in content, as content is fully counterbalanced across the experiment. Consequently, all that differs is familiarity or presumably, how engaging/distracting the TV content is.

**TABLE 1:** **Design of the experiment with each participant completing both sessions, and with episodes X, Y, and Z of Friends fully counterbalanced across participants**.

**Order of events**	**Same session**	**Different session**
1. Ratings I	Mood, hunger, fullness	Mood, hunger, fullness
2. Taste test I	Evaluate each snack food	Evaluate each snack food
3. Viewing only	**Episode X** of *Friends*	**Episode Y** of *Friends*
4. Evaluation I	Evaluate episode	Evaluate episode
5. Ratings II	Mood, hunger, fullness	Mood, hunger, fullness
6. Taste test II	Evaluate each snack food	Evaluate each snack food
7. Break	Find-a-word	Find-a-word
8. Viewing and snacking	Eat with **Episode X** of *Friends*	Eat with **Episode Z** of *Friends*
9. Evaluation II	Evaluate episode	Evaluate episode
10. Ratings III	Mood, hunger, fullness	Mood, hunger, fullness
11. Taste test III	Evaluate each snack food	Evaluate each snack food
12. Final session only (Three factor eating questionnaire, screen time eating/viewing habits)		

In addition to measuring food intake, the primary dependent variable, we also assessed mood, hunger and food palatability, during each session. This was to determine if these variables changed between sessions in a manner paralleling any alteration in food intake, because they all could potentially mediate the effects of TV (Brunstrom and Mitchell, 2006; Yeomans and Coughlan, 2009; Braude and Stevenson, 2014). Mood may be especially important, as content that is boring (possibly a repeated TV show) may generate negative affect, which participants may then attempt to mitigate by eating. TV viewing habits were also assessed as they have been shown to affect food intake (Braude and Stevenson, 2014). The three factor-eating questionnaire (Stunkard and Messick, 1985) was included because higher scores on one of its factors, dietary restraint, can sensitize participants to mood-induced eating (e.g., Yeomans and Coughlan, 2009). This could make more restrained individuals eat more in response to alterations in mood induced by TV. This would be important if mood changes were larger for a novel than for a repeated TV show. Finally, and as with several other studies in this area (Bellisle et al., 2004; Mittal et al., 2010; Ogden et al., 2013; Braude and Stevenson, 2014; Chapman et al., 2014) we used just female participants. This was based on considerations of power, as gender may moderate the effect of TV on eating behavior.

## Materials and Methods

### Participants

Forty-five female students (Mean age = 19.5, SD = 2.2, range 18–29; Mean BMI = 21.4, SD = 2.3, range 17.2–27.2) participated for course credit. All participants were telephone screened prior to testing to check that they had no food allergies or eating related problems (i.e., diabetes, special diets, psychiatric disorders). The study protocol was approved by the Macquarie University Human Research Ethics Committee and all participants consented to take part. The study was described to participants as exploring the role of environmental factors on food choice and a full debriefing was provided at the end regarding its specific aims. No participant reported being aware of the main prediction (i.e., greater food intake in the Same session).

### Design

A fully within-participant design (Different session vs. Same session) was used (See Table 1), with each participant attending the two sessions in counterbalanced order. On the Different session participants viewed a TV comedy show, followed by a second and different episode with snack food now available. On the Same session, participants viewed a further and different episode of the TV comedy show, and then watched *the same episode again*, with snack food now available. Crucially, allocation of the three episodes of the TV comedy show used here were fully counterbalanced across the experiment. This means that each episode was as likely to serve in the Same session as it was in the Different session, thus overall, equating content across sessions.

One potential problem with this design is that it presumes that participants will not previously have seen the episode used in the second part of the Different session (i.e., Episode Z in Table 1). Thus if participants were included here who had seen Episode Z before (see Table 1), this would obviate the basic purpose of the design to compare the effects of familiarity, while holding content constant. To address this problem of prior exposure, we asked participants if they had seen this (and the other) episode(s) before. Those who reported having seen the key episode (Z in Table 1) before were removed from the primary analysis.

### Materials

Three different popular snack foods were selected for this experiment; roasted almonds to appeal to more health conscious participants (Woolworths), original flavored Pringles chips (Kellogg’s Inc.,) and M&M’s (Mars Inc.,). Each food was presented individually during each taste test in a 25 ml disposable plastic sample cup and as an 80g portion in a clear plastic bowl (15 cm diameter) during each snack phase.

Three 20 min episodes of the popular TV comedy series “*Friends*” were used without breaks or advertisements: “The One with All the Rugby,” “The One with All the Resolutions,” and “The One with Rachel’s Inadvertent Kiss.” These episodes were chosen from Season 4 or 5 (released in 1998/1999) so that they would be of sufficient vintage to not have been seen by many participants. In addition, they contained no explicit references to eating and were not overtly emotive (as judged by the experimenters).

Two questionnaires were administered at the end of the second session. The first was the Three-Factor Eating Questionnaire (TFEQ; Stunkard and Messick, 1985), used primarily to measure dietary restraint (scores for disinhibition and hunger are also derived from the TFEQ). The second was a four item screen time habits measure, which asked: (1) how much TV (on any device) was watched per week (five-point scale from none to more than 15 h per week); (2) how much screen time other than TV was viewed per week on any device (five-point scale from none to more than 15 h per week); (3) how often eating occurred with TV (five point scale from never to more than once a day); and (4) how often eating occurred with other screen viewing activities (five point scale from never to more than once a day).

### Procedure

Participants were assigned by order of arrival to a predetermined counterbalancing schedule. This schedule dictated the order in which participants would complete the experiment (i.e., Same as session 1 and Different as session 2 or vice versa) and the particular episodes that would serve in each session for that participant. As session order did not influence the study outcomes, it is not further reported. Each session took around 1 h.

On the first session participants completed a brief questionnaire to obtain age, gender and any current medical condition, food allergies or intolerances, as well as what and when the participant had last eaten and drunk. Participants had been instructed in the telephone screen to refrain from eating and drinking energy/caffeinated beverages in the 2 h before the session, so as to increase the likelihood of snacking in the study. All reported complying with this request.

Participants were asked to report how happy, stressed, alert, relaxed, hungry and full they were using 15 cm line scales (anchors; Not at all and Extremely). These scales were repeated at various time points in each session and are referred to collectively as the Mood and Hunger Scales.

Participants then completed the first Taste Test, which involved consuming and evaluating one almond, one M&M and one Pringles chip, presented in counterbalanced order (all six possible orders were used for each participant, with these assigned randomly to each session, and time point within a session). After consuming each food item participants evaluated it on five 15 cm line rating scales: (1) Liking (anchors; Strongly Dislike, Indifferent and Strongly Like); (2) Frequency of consumption (anchors; Never and Very often); (3) Sweetness (Anchors; Not at all and Extremely); (4) Saltiness (Anchors; Not at all and Extremely); (5) Desire to consume more (Anchors; Do not want any more and Strongly desire more). This was followed by a ranking task with participants asked to rank, in order of preference, the three snack foods. Only the liking ratings were used in the analysis, as the other ratings were included to mask this primary focus (we note that the Desire ratings produced the same pattern of outcome as the reported Liking ratings).

The first TV episode was then viewed. Participants were asked to sit in a comfortable chair with a side table (all prior testing was completed at a desk) and were told, “You will now be asked to watch an episode of *Friends*. When the episode ends, let me know, I will be outside.” The participants were not given any snack foods during the first episode, but a cup of water was provided. Participants were asked to turn their mobile phone off and place their bag away from the TV viewing area. The researcher then left the room for the duration of the show.

After the episode ended the participant was asked to move back to the desk and to evaluate the show. Participants were asked to briefly describe the episode they had just watched, to indicate whether they had seen the episode before at home or elsewhere (but explicitly *not* referring to viewing in the laboratory) and to rate their liking for the episode on a 15 cm line scale (anchors; Strongly Dislike, Indifferent and Strongly Like). This was immediately followed by the second administration of the Mood and Hunger scales, and then by the second Taste Test.

The participant was then given a 5-min distraction task, which involved completing two word searches. This allowed the researcher time to prepare two snack food bowls, one containing 80 × *g* of their most preferred snack food and one containing 80 × *g* of their second most preferred snack food, as rated by the participant in their second Taste Test. Two foods were used so as to provide variety, as a single food might engender monotony and place a ceiling on intake. The two snack bowls and another cup of water were then placed on the table beside the chair facing the TV. As there were no differences in the amount drunk between sessions, water intake is not reported.

Once the 5-min interval was completed, participants were brought back to the TV viewing area and were told: “You will now be asked to watch the same (or a different) episode of *Friends* and you have been given snack foods to eat while watching the episode. Please eat as much as you like as the uneaten food will be thrown away.” The researcher again left the room and the participant watched either the same or a different episode of *Friends*.

After the second episode ended, participants were again seated at the desk to complete a further set of tasks: (1) evaluate the TV episode as described above; (2) complete the third administration of the Mood and Hunger scales; and (3) complete the third Taste Test. This concluded the first session. Once the participant had left the room, the quantity of food consumed and water drunk was recorded.

Participants returned 1 week later for their second session. Participants were asked to record what they had eaten and drunk prior to the session, and whether they had again adhered to the instructions to avoid eating in the 2 h preceding the study (all reported that they had). The procedure for the second session was identical to the first—including providing the same participant-specific snack foods—except for two things. First, if participants had watched the same episode of “*Friends*” twice in Session One (i.e., the Same session), then they watched two different episodes in Session Two (i.e., the Different session), and vice versa. Second, at the end of the second session, participants completed the TFEQ and the screen time habits questionnaire. Participants were then weighed and measured, so as to calculate BMI.

### Analysis

In the Different session, 11 of the 45 participants had seen the key second episode before (i.e., episode Z in Table 1). These 11 participants were excluded from the primary analysis as they were familiar with this episode. The episode counterbalancing for the remaining 34 participants was unaffected by these exclusions (i.e., distribution of episodes to conditions (i.e., X, Y, Z in Table 1) was almost exactly equal).

In the Same session, 13 participants had seen the repeated episode before (i.e., episode X in Table 1). Eight of these 13 had also seen the key second episode in the Different session before as well (i.e., episode Z in Table 1). As having seen the Same session episode before (i.e., episode X in Table 1) should not affect the predicted outcomes (i.e., episode X was repeated here anyway), the 5 participants who had seen the repeated episode before were retained. We note that if all 16 participants who had seen either episode X or Z before are removed, the reported outcomes are the same as described for the primary analyses below.

As the quantity of food consumed and the liking data were not normally distributed, non-parametric tests were used for these variables. While we report test results for only the quantity of food consumed, we note that the same significance levels are achieved when energy content or number of items eaten serve as the unit of measurement. Liking data from the first Taste test is not reported (there were no differences by Same/Different session in liking ratings for the three snack foods) as this test merely served to ensure that events surrounding both viewing components of each session were kept as similar as possible.

## Results

### The Effect of TV Content Familiarity on Snack Food Intake

Food intakes (grams consumed and energy) for the Same and Different session and for the most preferred and second most preferred snack foods, are presented in Table 2. Overall, participants consumed significantly more snack food in the Same session (*M* = 76.2g, SD = 36.2) than in the Different session (*M* = 66.7g, SD = 37.3), Wilcoxon test, *Z* = 1.90, *p* < 0.05 (one tailed). This represents a mean additional energy intake of 211 KJ or around 2% of a sedentary adults daily energy intake (*circa* 9000 KJ).

**TABLE 2:** **Mean (and standard deviation) food intake (in grams and kilo Joules) during the study by condition (Same vs. Different) and food type (most and second most preferred snack food)**.

**Intake measure**
	**Same condition**		**Different condition**		**Difference (Same–Different)**	
	**First Pref^1^**	**Second Pref^1^**	**First Pref^1^**	**Second Pref^1^**	**First Pref^1^**	**Second Pref^1^**
g	46.3 (22.8)	30.0 (18.6)	39.1 (20.4)	27.6 (21.5)	7.2 (22.1)	2.3 (18.7)
KJ	1005.2 (503.9)	669.4 (404.6)	842.0 (432.0)	622.1 (478.7)	163.2 (483.9)	47.3 (429.0)

^1^First Pref is the participants most preferred snack food and Second Pref is their second most preferred snack food.

Participants were given two snack foods to eat, their most and second most preferred choice. Examination of Table 2 suggests that more was eaten of the most preferred food, which is not surprising as it was given a consistently (across all tests) higher liking rating (*M* = 128.4/150) than the second most preferred food (*M* = 112.6/150), Wilcoxon test, *Z* = 3.77, *p* < 0.001. We tested if food intake for the most preferred and second most preferred snack food, differed across sessions. For the most preferred snack food, participants ate significantly more in the Same session relative to the Different session, Wilcoxon test, *Z* = 2.55, *p* < 0.01. There was no significant difference for the second most preferred food.

Recall that data from 11 participants were excluded as they had seen the second Different episode before (i.e., episode Z in Table 1). We tested one secondary *post hoc* prediction using these data (note that the remaining analyses after this use just the 34 participants data). We contrasted the difference in food intake between the Same and Different sessions for the 34 participants where TV differed in familiarity, against the 11 participants where it did not [i.e., episode Z and X in Table 1 were both familiar; note that there was no difference in food intake between sessions for these 11 participants, *Z* = 1.3 (Different *M* = 91.2, SD = 45.9; Same *M* = 71.9, SD = 51.6)]. A Mann-Whitney U-test indicated that significantly more food was consumed in the Same session relative to the Different session in the 34 participants for whom the TV shows differed in familiarity (*M* difference = 9.5, SD = 34.4) than for the 11 participants with no difference in familiarity (*M* difference = –19.3, SD = 39.9), *Z* = 2.03, *p* < 0.05. This further suggests that episode familiarity affects food intake.

### Changes in Liking for the Snack Foods

Liking scores for the Same and Different sessions and snack food types are presented in Table 3. We started by testing if the change in liking for the most preferred food was larger for the Same session relative to the Different session. There was no difference in the magnitude of the change of liking across sessions. This suggests that although participants ate more of their most preferred food during the Same session, they did not show a larger reduction in liking for it.

**TABLE 3:** **Mean (and standard deviation) liking for each food type (most and second most preferred snack food) by condition (two identical episodes—Same vs. two different episodes—Different) across the experiment**.

**Condition Variable**	**Mean (SD)**
(A) Same condition	
1. Pre-snack, first preference	124.8 (17.0)
2. Pre-snack, second preference	118.4 (16.6)
3. Post-snack, first preference	113.9 (18.3)
4. Post-snack, second preference	100.8 (23.9)
5. Difference (A1–A3), first preference	10.9 (20.4)
6. Difference (A2–A4), second preference	17.6 (21.6)
(B) Different condition	
1. Pre-snack, first preference	124.3 (13.2)
2. Pre-snack, second preference	113.1 (18.2)
3. Post-snack, first preference	114.0 (20.8)
4. Post-snack, second preference	106.6 (27.8)
5. Difference (B1–B3), first preference	10.4 (18.9)
6. Difference (B2–B4), second preference	6.5 (23.5)
(C) Same minus Different condition	
1. Difference (A5–B5), first preference	0.5 (18.5)
2. Difference (A6–B6), second preference	11.1 (30.1)

We then repeated this analyses for the second most preferred food. There was a significantly larger reduction of liking in the Same session than in the Different session for the second most preferred food, *Z* = 2.30, *p* < 0.02. In this case, although changes in liking were greater in the Same session, this was not accompanied by any difference in food intake.

### Changes in Hunger and Fullness

Hunger ratings were analyzed using a repeated measures ANOVA, with Time (Start of the study vs. pre-eating vs. post-eating) and Session (Same vs. Different) as within factors. The ANOVA revealed just one significant effect, that of Time, with hunger ratings initially increasing across the course of the experiment, *F*(2,66) = 18.48, *p* < 0.001, ${\text{η}}_{p}^{2}$ = 0.36, from a mean of 71.6–84.1 and then falling after consumption of the snack food to 46.9.

Fullness ratings were analyzed using the same ANOVA design. They too revealed a main effect of Time, *F*(2,66) = 17.55, *p* < 0.001, ${\text{η}}_{p}^{2}$ = 0.35, with fullness ratings initially falling from the start of the experiment (*M* = 56.4) to the pre-eating rating (*M* = 48.5) and then increasing after snacking (*M* = 81.6). There were no other significant main effects or interactions.

### Changes in Happiness, Stress, Alertness, and Relaxation

Each rating type was analyzed using repeated measures ANOVA, with Time (Start of the study vs. pre-eating vs. post-eating) and Session (Same vs. Different) as within factors.

Happiness ratings significantly changed across factor Time, *F*(2,66) = 7.72, *p* < 0.001, ${\text{η}}_{p}^{2}$ = 0.19, from a mean of 95.3–105.3 prior to the snack food and then to a mean of 104.3 after the snack food. There were no other significant effects.

Stress ratings significantly decreased across factor Time, *F*(2,66) = 8.96, *p* < 0.001, ${\text{η}}_{p}^{2}$ = 0.21, from a mean of 43.2–31.5 prior to the snack food and then to a mean of 32.1 after the snack food. There were no other significant effects.

There were no significant effects involving alertness ratings. Relaxation ratings significantly changed across factor Time, *F*(2,66) = 11.07, *p* < 0.001, ${\text{η}}_{p}^{2}$ = 0.25, from a mean of 86.8–101.7 prior to the snack food and then to a mean of 103.3 after the snack food. There were no other significant effects.

### Evaluation of the TV Programs

Participants evaluated how much they liked each TV show after the program had finished. These ratings were analyzed using a repeated measures ANOVA, with Time (First show vs. Second show) and Session (Same vs. Different) as within factors. The ANOVA revealed an interaction of Session by Time, *F*(1,33) = 13.54, *p* < 0.001, ${\text{η}}_{p}^{2}$ = 0.29. The liking means indicate the source of this interaction, with no significant change in liking ratings in the Different session (*M* for first show = 118.7; *M* for second show = 123.2; *p* = 0.11), but a significant reduction in liking ratings in the Same session [*M* for the first show = 121.0; *M* for the second (repeated) show = 112.8; *p* < 0.02]. There were no other significant effects.

### Prior Screen Time Eating and Viewing Habits

Participants spent a median of 6–10 h TV viewing per week and a median of 11–15 h viewing other electronic devices (smart phones, tablets etc). They also reported eating in front of the TV a median of a few times per week, as with eating in front of other electronic devices. There were positive associations between TV viewing and TV eating, Spearman’s rho = 0.57, *p* < 0.001, and screen viewing and screen eating, Spearman’s rho = 0.37, *p* < 0.05. There were no significant associations between any of the screen time habit variables and total food consumed across both sessions nor between the Same and Different sessions (or by food type).

### Three-factor Eating Questionnaire

Participants had a mean Restraint score of 8.7 (range 0–19, SD = 5.4), a mean Disinhibition score of 6.7 (range 2–15, SD = 3.6) and a mean Hunger score of 5.8 (range 1–12, SD = 3.2). There were no significant associations between Restraint scores and the amount of food eaten during the experiment (overall, nor difference between sessions or by food type, nor by moderation of mood).

## Discussion

TV viewing can exert a number of effects on energy balance (e.g., Landhuis et al., 2008; Dunstan et al., 2010; Wijndaele et al., 2010). Several studies have observed that eating with TV can result in increased intake (e.g., Blass et al., 2006; Hetherington et al., 2006; Braude and Stevenson, 2014), although this has not been uniformly observed (e.g., Martin et al., 2009; Peneau et al., 2009; Chapman et al., 2014). One possible reason for this variability in outcome—as discussed in the Introduction—may be the degree of engagingness (or distraction) afforded by what is being viewed (e.g., Brunstrom and Mitchell, 2006; Moray et al., 2007; Ogden et al., 2013). The claim that varying levels of distraction might have different effects on food intake have mainly been inferred from studies that manipulated either tasks (e.g., TV vs. driving) or TV content (e.g., comedy vs. boring). One concern with drawing inferences from such designs is that they confound content with its capacity to distract/engage. In the experiment reported here we attempted to test the impact of distraction/engagement by manipulating content familiarity. In the Same session participants viewed the same episode of *Friends* twice, with snack food presented on the second viewing. In the Different session they viewed two different episodes of *Friends*, again with snack food present with viewing the second episode. Participants who conformed to the design, namely those who had not previously seen the second episode before in the Different session, demonstrated the predicted effect. They ate significantly less snack food when watching a novel episode of *Friends* than they did when viewing a familiar episode. In addition, this difference in food intake also significantly exceeded that of the 11 excluded participants who were familiar with both episodes (i.e., who had seen the second episode before in the Different session). This suggests that a novel TV comedy reduces food intake, relative to a familiar TV comedy.

One possible interpretation of our findings is that the repeated episode was less distracting than the novel episode. As a result, participants may have been more able to divide their time between eating and viewing, rather than spending (presumably) more time watching the novel episode at the expense of eating. However, this is not the only way that this result could be interpreted. Two other possibilities can be envisaged. As Chapman et al. (2014) suggest, inducing boredom may in and of itself trigger eating. As an immediately repeated episode is likely to be more boring than a novel episode, differences in food intake could be attributed to greater boredom even in the absence of any negative mood state. As we did not measure boredom, it is plausible that this accounts for the excess energy intake, however, the finding that the repeated TV show was still judged as liked would seem inconsistent with it also being judged as boring.

A second possibility is that the repeated presentation of the same TV show adversely affected mood. Mood is known to significantly affect food intake (e.g., Groesz et al., 2012; Bongers et al., 2013) and dietary restraint can moderate this effect (Yeomans and Coughlan, 2009). There were no significant differences on any of the mood variables between the Same and Different session, suggesting that changes in mood were unlikely to account for differences in intake. Moreover, dietary restraint as measured by the TFEQ (Stunkard and Messick, 1985) was not associated with differences in food intake, nor did it moderate moods relationship with this variable. A further and related possibility is that some unmeasured aspect of mood state—notably irritation—might have driven greater food intake on the “Same” session. This is plausible, as participants could have felt annoyed and irritated when asked to view the same episode of *Friends* again. While we did not measure irritation *per se*, and so cannot know for sure, any increased irritability would *probably* impact the other mood ratings. So, while irritation (and boredom) *could* account for the food intake effects observed in this study, there are some plausible objections to each of these accounts.

While our primary analysis focussed just on the participants who had not seen the novel episode before in the Different session (i.e., episode Z), we also conducted a secondary analysis comparing their food intake with that of the 11 participants for whom the novel episode was in fact familiar. This comparison indicated a different pattern of food intake between these two groups, with the anticipated outcome (i.e., significantly more being eaten in the familiar Same session) being observed in the bulk of participants who conformed to the design. In the remaining 11 participants, food intake tended to be higher in the Different session, noting that this effect was not reliable. This is interesting, as one might have expected intake on the Different and Same sessions to be nearly identical here, as both the Different and Same session second episodes were familiar. However, what makes interpretation problematic here—and indeed drawing any firm conclusion—is that 8/11 participants had also seen the episode that was repeated in the Same session before as well.

We suggested that distraction might also account for the observed differences in food intake. This could occur via several means. A TV show could be so engrossing that people might forget to eat. Engagingness could modulate eating rate, or the length of the intervals between bouts. It could also affect attention to cues that might normally signal the end of a meal, such as an empty or emptying bowl, a sense of fullness, reduced hunger or reduced liking for the food. While there is evidence to suggest that some of these factors can play a role in TV’s effect on food intake (e.g., Brunstrom and Mitchell, 2006; Braude and Stevenson, 2014), we found little evidence for them here. No session related changes in hunger or fullness were observed, and the only variable that changed across sessions was liking for the second most preferred food. This decreased to a greater extent in the Same session than in the Different session. This was unexpected, as one would have predicted that the most preferred food where intake differed most between sessions, would be most likely to demonstrate differential changes in liking.

Apart from not measuring boredom or irritation, two further limitations of the design warrant mention. The first is while content is controlled here via counterbalancing, strictly speaking we can only say that TV familiarity affects intake when the genre is comedy. Whether familiarity exerts similar effects on other genres remains to be established. The second is the absence of a no TV control. While we can know that the relative familiarity of two comedy shows can affect food intake, we cannot know how these stand relative to a baseline no TV control. Thus we do not know if the novel show (Different session) enhances intake, has no effect or even depresses intake relative to a no TV condition. The absence of no TV condition might also explain the failure to obtain any correlation between food intake in this study and the TV viewing habits questionnaire. We previously observed an association between frequency of eating with TV and the magnitude of the effect of TV on eating during an experiment (i.e., amount eaten with TV minus amount eaten with no TV; Braude and Stevenson, 2014). It is possible that our failure to observe an association here is a consequence of not being able to estimate the effect of TV *per se* on food intake due to the absence of no TV control. Moreover, we note that the questions used to assess eating with TV do not indicate whether eating *always* accompanies TV viewing or is occasional, which is likely to be important (i.e., whether there is a contingent relationship).

In this study we have demonstrated that varying familiarity of a TV comedy can affect food intake, with more eaten with a familiar than with a novel episode. As episodes were counterbalanced across participants, this effect cannot be attributed to differences in the content of the episodes for this genre. We suggest that this effect may reflect how engaging the TV is, that is the extent to which it distracts from eating. It is not clear how this putative distraction effect works, and it is also apparent that TV can affect food intake through several other routes in addition to distraction, such as via irritability and boredom.

### Conflict of Interest Statement

The authors declare that the research was conducted in the absence of any commercial or financial relationships that could be construed as a potential conflict of interest.
